# Extracellular calcium increases fibroblast growth factor 2 gene expression via extracellular signal-regulated kinase 1/2 and protein kinase A signaling in mouse dental papilla cells

**DOI:** 10.1590/1678-7757-2017-0231

**Published:** 2018-05-03

**Authors:** Sousuke Kanaya, Binlu Xiao, Yukihiko Sakisaka, Mizuki Suto, Kentaro Maruyama, Masahiro Saito, Eiji Nemoto

**Affiliations:** 1 Tohoku University, Graduate School of Dentistry, Department of Periodontology and Endodontology, Sendai, Japan; 2 Tohoku University, Graduate School of Dentistry, Liaison Center for Innovative Dentistry, Sendai, Japan; 3 Tohoku University, Graduate School of Dentistry, Department of Restorative Dentistry, Division of Operative Dentistry, Sendai, Japan

**Keywords:** Mouse dental papilla cells, Extracellular calcium, Fibroblast growth factor 2, Bone morphogenetic protein 2

## Abstract

We previously reported that elevated extracellular calcium (Ca^2+^) levels increase bone morphogenetic protein 2 expression in human dental pulp (hDP) cells. However, it is unknown whether extracellular Ca^2+^ affects the expression of other growth factors such as fibroblast growth factor 2 (FGF2).

**Objective:**

The present study aimed to examine the effect of extracellular Ca^2+^ on FGF2 gene expression in hDP and immortalized mouse dental papilla (mDP) cells.

**Materials and Methods:**

Cells were stimulated with 10 mM CaCl_2_ in the presence or absence of cell signaling inhibitors. FGF2 gene expression was assessed using real-time polymerase chain reaction. The phosphorylation status of signaling molecules was examined by Western blotting.

**Results:**

Extracellular Ca^2+^ increased FGF2 gene expression in mDP and hDP cells. Gene expression of the calcium-sensing receptor and G protein-coupled receptor family C group 6 member A, both of which are extracellular Ca^2+^ sensors, was not detected. Ca^2+^-mediated *Fgf2* expression was reduced by pretreatment with the protein kinase A (PKA) inhibitor H-89 or extracellular signal-regulated kinase (ERK) 1/2 inhibitor PD98059 but not by pretreatment with the protein kinase C inhibitor GF-109203X or p38 inhibitor SB203580. Extracellular Ca^2+^ increased PKA activity and ERK1/2 phosphorylation. Ca^2+^-induced PKA activity decreased by pretreatment with PD98059.

**Conclusions:**

These findings indicate that elevated extracellular Ca^2+^ levels led to increased *Fgf2* expression through ERK1/2 and PKA in mDP cells and that this mechanism may be useful for designing regenerative therapies for dentin.

## Introduction

Dental pulp is a dental papilla-derived mesenchymal tissue. Dental papilla and dental pulp contain cranial neural crest- and mesoderm-derived cells, and cranial neural crest-derived pluripotent stem cells are capable of differentiating into odontoblasts and producing primary dentin^4^. Dental follicle cells have the same origin as dental papilla cells, and they are capable of differentiating into fibroblasts, osteoblasts, and cementoblasts. During tooth root development, matrix molecules secreted by the epithelial root sheath of Hertwig may differentiate dental papilla and dental follicle cells into odontoblasts and cementoblasts, respectively^5^.

Dentin is formed via the odontoblast-induced mineralization of collagenous substrates. Calcium (Ca^2+^)-based materials, such as Ca(OH)_2_ and mineral trioxide aggregate (MTA), have been used in direct or indirect pulp capping treatment^28^. Recently, An, et al.^1^ (2012) reported that extracellular Ca^2+^ promotes osteogenic differentiation and mineralization in human dental pulp (hDP) cells. We previously reported that elevated extracellular Ca^2+^ levels lead to increased bone morphogenetic protein 2 (BMP2) gene expression in hDP cells^26^. BMP2, which is a crucial regulator of osteogenic differentiation and odontoblastic differentiation, promotes dentin formation *in vitro* and *in vivo* ^11^. Moreover, Li, et al.^16^ (2015) reported that extracellular Ca^2+^-induced BMP2 promotes the odontogenic differentiation of dental pulp stem cells via extracellular signal-regulated kinase (ERK) 1/2 pathways, indicating that extracellular Ca^2+^ promotes dentin regeneration.

Pulp capping materials exhibit various solubility and Ca^2+^ release profiles. Among these, MTA releases high Ca^2+^ concentrations. Takita et al. reported that MTA released approximately 0.3 mM Ca^2+^ and significantly increases hDP cell proliferation^27^. Under physiological conditions, the Ca^2+^ concentration in the predentin area reaches as high as 35 mM^18^. In the present study, mDP and hDP cells were stimulated with 10 mM extracellular Ca^2+^, which induced an effective cellular response.

Fibroblast growth factor (FGF) 2, which is a potent regulator of the growth, survival, and differentiation of mesenchymal cells, plays an important role in bone formation and remodeling^17^. FGF2 promotes the proliferation of preosteoblasts, which can differentiate into mature osteoblasts, and the proliferation of hDP cells by inhibiting alkaline phosphatase activity^24^. By contrast, FGF2 can stimulate the expression and transactivation activity of runt-related transcription factor 2, which can facilitate osteoblast differentiation^14^. These reports have indicated that the effect of FGF2 on proliferation and differentiation depends on the cell origin or differentiation stage. Sagomonyants, et al.^23^ (2015) found that the early and limited exposure of dental pulp cells to FGF2 significantly increases the expression of dentin sialophosphoprotein, which is an odontoblast differentiation marker, indicating that FGF2 stimulates dental pulp cell differentiation and promotes dentin regeneration. Although many studies have reported the effect of FGF2 on cellular response, the possible induction of FGF2 expression in cells has not been fully investigated. In the present study, we found that elevated extracellular Ca^2+^ levels increased FGF2 gene expression in hDP cells, mouse dental papilla (mDP) cells, and a mouse dental follicle cell line (SVF4); we analyzed the mechanisms underlying the increase in FGF2 gene expression in mDP cells.

## Materials and methods

### Cell culture

mDP cells, which were immortalized via the expression of the mutant human papillomavirus type 16 E6 gene lacking the PDZ-domain-binding motif, were used^2^. Cells were maintained in Dulbecco's modified Eagle's medium (DMEM) (Gibco BRL Rockville, MD, USA) containing 10% heat-inactivated fetal bovine serum (FBS) (Gibco), 100 U/ml penicillin G, and 100 μg/ml streptomycin. hDP cells were obtained from the extracted third molars of healthy individuals (19–29 years old) at Tohoku University Hospital with informed consent. A groove was made in the buccal and occlusal tooth surfaces in a buccolingual direction using a dental fissure bur. Teeth were split using tooth forceps and a chisel. Dental pulp tissue was separated from teeth, cut into small pieces, and then cultured in α-minimum essential medium (α-MEM) (Gibco) containing 10% FBS and antibiotics, with the medium changed every 3 days until subconfluent (70–80% confluent) cell monolayers were formed. After reaching confluency, cells were passaged with a solution containing trypsin (0.25%) and ethylenediaminetetraacetic acid (0.1%). Dental pulp cells from subconfluent monolayers at subculture levels 3–9 were used in the experiments. Experimental procedures were approved (approval number: 26–27) by the Ethical Review Board of Tohoku University Graduate School of Dentistry (Sendai, Japan). SVF4, an immortalized with SV40 mouse dental follicle cell line^30^, was kindly provided by Dr. Martha J. Somerman (National Institute of Dental and Craniofacial Research, Bethesda, MD, USA) and maintained in DMEM containing 10% FBS and antibiotics. All tissue culture reagents were purchased from Invitrogen/Gibco BRL (Carlsbad, CA, USA).

### Reverse transcription and real-time quantitative polymerase chain reaction (PCR)

**Figure 1:**
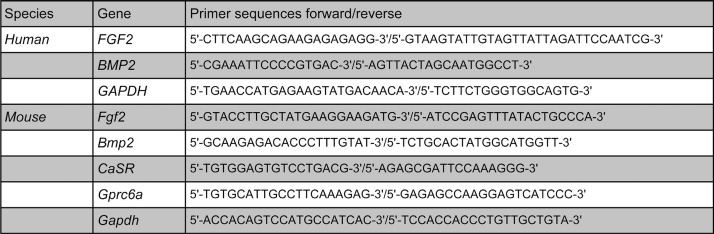
Primer sequence used for polymerase chain reaction amplifications

As the basal Ca^2+^ concentration in the medium was 1.8 mM, anhydrous CaCl_2_ (Wako Industries, Ltd, Osaka, Japan) was added to the cell culture to obtain a final Ca^2+^ concentration of 10 mM. Cells were plated on 6-well multi-plates and then stimulated with 10 mM CaCl_2_ or MgCl_2_ (Sigma Chemical Co., St. Louis, MO, USA). Before 6 h of stimulation, a subconfluent monolayer of mDP or SVF4 cells was serum-deprived for 4 h in DMEM. A subconfluent monolayer of hDP cells was serum-deprived for 4 h in α-MEM. To assess the involvement of intracellular signaling, cells were pretreated with 1 μM GF109203X [protein kinase C (PKC) inhibitor], 10 μM H-89 [protein kinase A (PKA) inhibitor, Sigma], 10 μM SB203580 (p38 inhibitor), or 10 μM PD98059 (ERK1/2 inhibitor, Calbiochem, Darmstadt, Germany) for 30 min and were then incubated for 6 h with 10 mM CaCl_2_. All treatments used an equal concentration of 0.05% (v/v) dimethyl sulfoxide (Wako Pure Chemical Industries, Ltd., Osaka, Japan). Total cellular RNA was extracted using Qiashredder and RNeasy® Kits (QIAGEN, Valencia, CA, USA) according to the manufacturer's instructions and was treated with DNase (DNA-free™, Ambion Inc., Austin, TX, USA). The transcription of total RNA into cDNA was performed using Transcriptor First Strand cDNA Synthesis Kit® (Roche Diagnostic Co., Indianapolis, IN, USA) according to the manufacturer's instructions. Mouse kidney cDNA was purchased from Clontech Takara Bio Co. (Shiga, Japan). Primers were designed using LightCycler probe design software® (Roche Diagnostics GmbH, Mannheim, Germany), and primer sequences for each gene are described in Figure 1. The amplification profile was 40 cycles *temperature [°C]/time [s]) of 95/60, 55/30, and 72/30. PCR was performed in iCycler® (Bio-Rad Laboratories, Hercules, CA, USA) using iQ SYBR Green Supermix® (Bio-Rad) with optimized levels of 3 mM MgCl_2_ and 0.5 μM of each primer. After amplification, one cycle of the linear temperature gradient from 55°C to 95°C at a transition rate of 0.5°C/30 s was employed to assess the specificity of the PCR products. In each run, water was used as the negative control. Reaction products were quantified using glyceraldehyde 3-phosphate dehydrogenase as the reference gene. For end-point PCR, the amplification profile was 35 cycles using the same cycle program (temperature/time). Amplified samples were visualized on 2.0% agarose gels stained with ethidium bromide and photographed under ultraviolet light. Multiple tissue cDNA panels were purchased from BD Biosciences Clontech (Palo Alto, CA, USA).

### PKA activity

PKA activity was measured using PKA Kinase Activity Kit (Enzo Life Sciences, Inc, Farmingdale, NY, USA) according to the manufacturer's instructions. A subconfluent monolayer of cells cultured in a 6-well multi-plate was serum-deprived for 4 h in DMEM and then stimulated with 10 mM CaCl_2_ for the indicated times at 37°C. The medium was removed, and 150 μl of 20 mM Tris-HCl containing 1% (v/v) Triton was added to the wells. After 10 min of incubation on ice, the lysate was collected and centrifuged for 15 min at 15,000 ×*g*. The supernatant was collected and stored at −70°C until further use.

### Western blot analysis

A subconfluent monolayer of cells was serum-deprived for 4 h in DMEM and then stimulated with 10 mM CaCl_2_. Cells were harvested with Cell Lysis Buffer® (Cell Signaling, Beverly, MA, USA) according to the manufacturer's instructions. Cell lysates were separated by sodium dodecyl sulfate-polyacrylamide gel electrophoresis and transferred to a polyvinylidene difluoride membrane (ATTO, Tokyo, Japan). Membranes were blocked for 1 h in 5% (w/v) non-fat dried milk in phosphate-buffered saline with 0.1% (v/v) Tween 20 and incubated with anti-phosphorylated p38^Thr180/Tyr182^, anti-p38, anti-ERK1/2, or anti-phosphorylated ERK1/2^Thr202/Tyr204^ (all Cell Signaling) at a 1:1000 dilution at room temperature for 1 h. Blots were washed and incubated with horseradish peroxidase-conjugated secondary antibodies (Cell Signaling) at a 1:2000 dilution at room temperature for 1 h. Proteins were detected using ECL Prime Western blotting detection reagents (GE Healthcare, Madison, WI, USA) according to the manufacturer's instructions and visualized using Molecular Imager® ChemiDoc™ XRS plus (Bio-Rad).

### Statistical analysis

All experiments were performed in triplicate to test the reproducibility of the results, and representative findings are shown. Experimental values are denoted as mean ± SD. The significance of differences between the control and treatment groups was evaluated by Student's *t*-test or one-way analysis of variance. *P-*values less than 0.05 were considered significant.

## Results

### Elevated extracellular Ca^2+^ concentrations increased the expression of *Fgf2* and *Bmp2* in dental pulp cells

**Figure 2:**
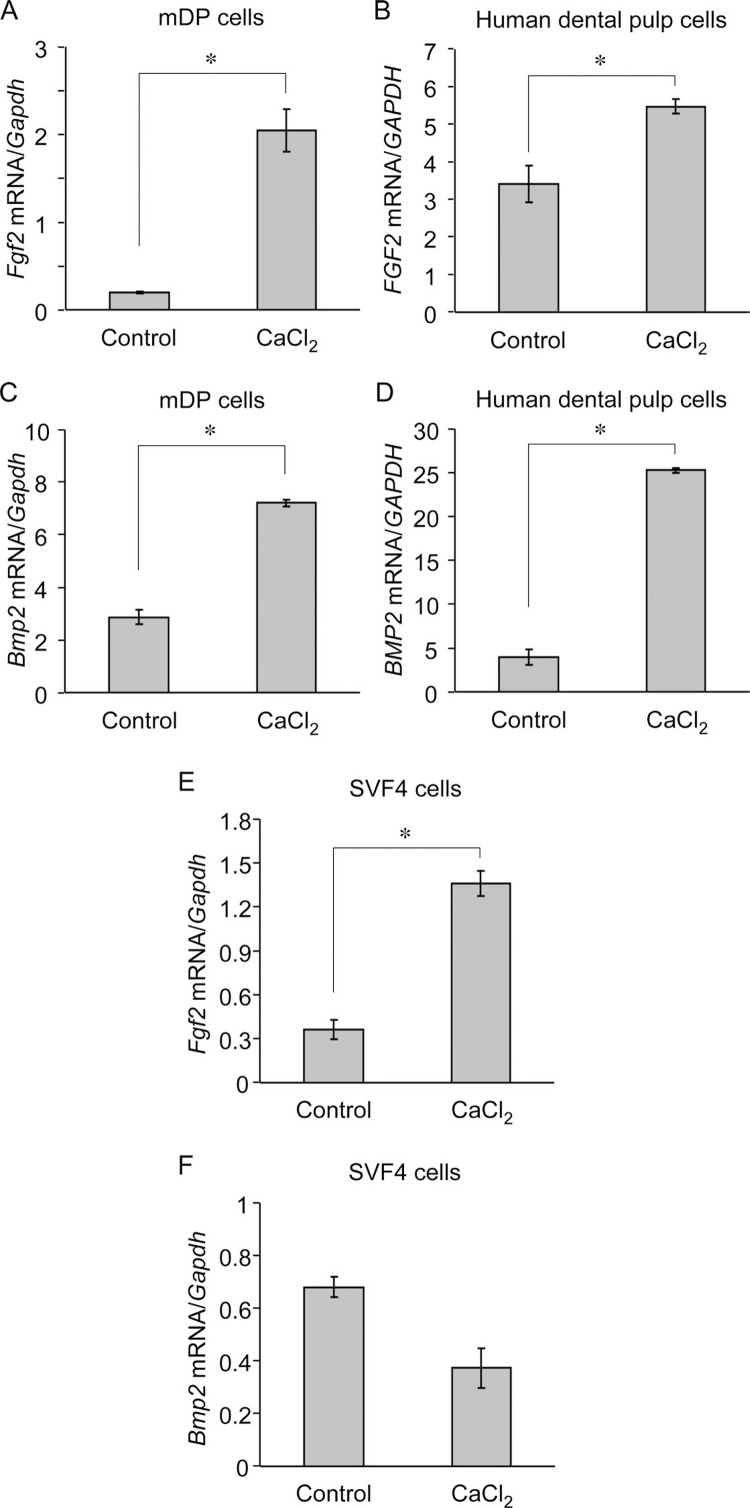
Stimulation of mouse dental papilla (mDP) and dental pulp cells with 10 mM CaCl_2_ increased fibroblast growth factor 2 (*Fgf2*) and bone morphogenetic protein 2 (*Bmp2*) expression. Subconfluent monolayers of cells were serum-deprived for 4 h. (A–F) mDP, human dental pulp, and mouse dental follicle (SVF4) cells were stimulated with 10 mM CaCl_2_ for 6 h in a serum-free medium. *Fgf2* and *Bmp2* expression was assessed using real-time reverse transcription-polymerase chain reaction. Representative data from three separate experiments are shown as the mean ± SD of triplicate assays. *, *P*<0.05 versus untreated cells using Student's *t*-test.

We found that stimulation with 10 mM CaCl_2_ for 6 h increased *Fgf2* expression in mDP (Figure 2A) and hDP (Figure 2B) cells. Similar results were observed in hDP cells derived from four different donors (data not shown). In addition, we found that stimulation with 10 mM CaCl_2_ increased *Bmp2* expression in mDP cells (Figure 2C). The increase in BMP2 levels was reproduced in Ca^2+^-stimulated hDP cells as we previously reported^26^ (Figure 2D). Elevated extracellular Ca^2+^ levels also resulted in increased expression of *Fgf2*, but not of *Bmp2*, in SVF4 cells (Figures 2E and F).

### Lack of involvement of calcium-sensing receptor (CaSR) and G protein-coupled receptor family C group 6 (GPRC6A) in extracellular Ca^2+^-increased *Fgf2* expression in mDP cells

**Figure 3:**
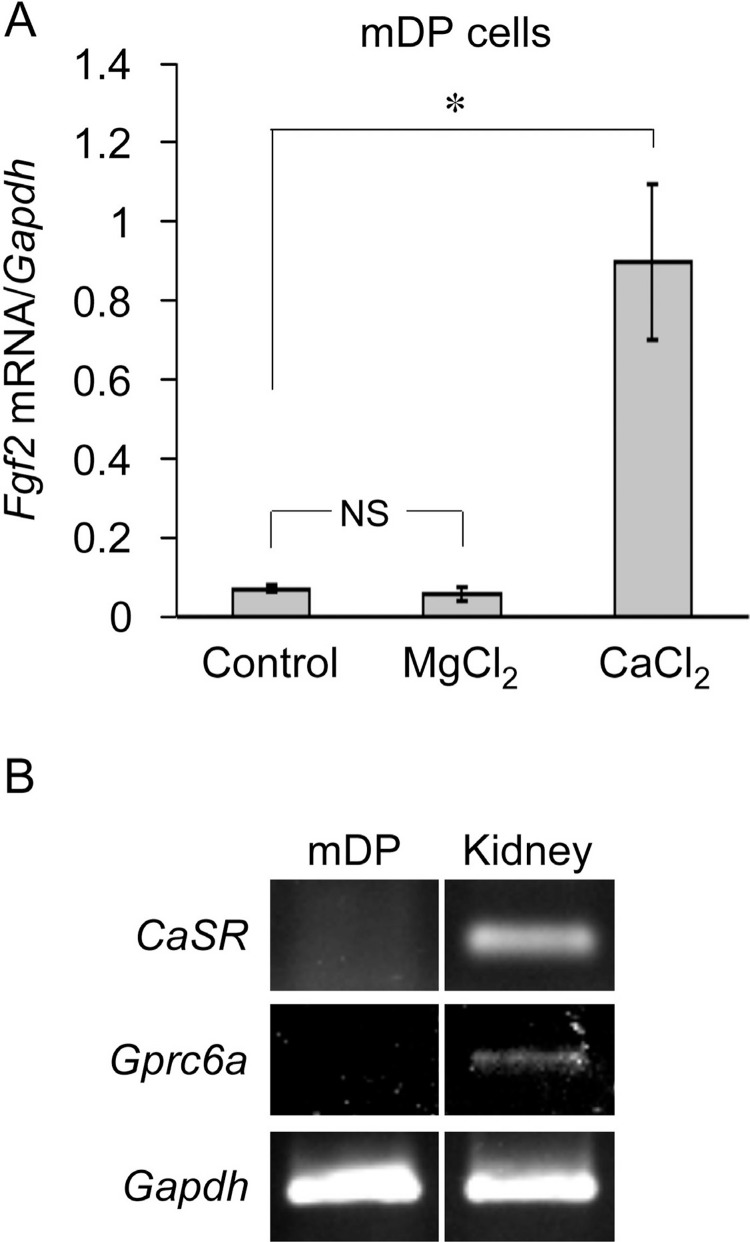
Elevated extracellular Ca^2+^-increased fibroblast growth factor 2 (*Fgf2*) expression is not mediated through calcium-sensing receptor (CaSR) or G protein-coupled receptor family C group 6 (GPRC6A) in mouse dental papilla (mDP) cells. (A) A subconfluent monolayer of mDP cells was serum-deprived for 4 h and then stimulated with 10 mM MgCl_2_ or CaCl_2_ for 6 h in a serum-free medium. (B) *CaSR* and *Gprc6a* expression in mDP cells was analyzed using reverse transcription-polymerase chain reaction (RT-PCR). Template cDNA from the murine multi-tissue cDNA library (kidney) was used as a positive control. *Fgf2* expression was analyzed using real-time RT-PCR. Representative data from three separate experiments are shown as the mean ± SD of triplicate assays. *, *P*<0.05 versus untreated cells using Student's *t*-test; NS, not significantly different from untreated cells.

We examined the ligand specificity of the receptors expressed by mDP cells. *Fgf2* expression increased upon stimulation with 10 mM CaCl_2_ but not with 10 mM MgCl_2_ (Figure 3A), indicating that the receptor in mDP cells is selective for Ca^2+^. We examined whether CaSR^21^ and GPRC6A^20^, both of which have sensitivity for both Ca^2+^ and divalent cations such as Mg^2+^, were expressed by mDP cells using reverse transcription-PCR. The genes encoding CaSR and GPRC6A were detected in a brain cDNA library as positive controls but not in mDP cells (Figure 3B).

### Extracellular Ca^2+^ increased *Fgf2* expression via the PKA pathway in mDP cells

**Figure 4:**
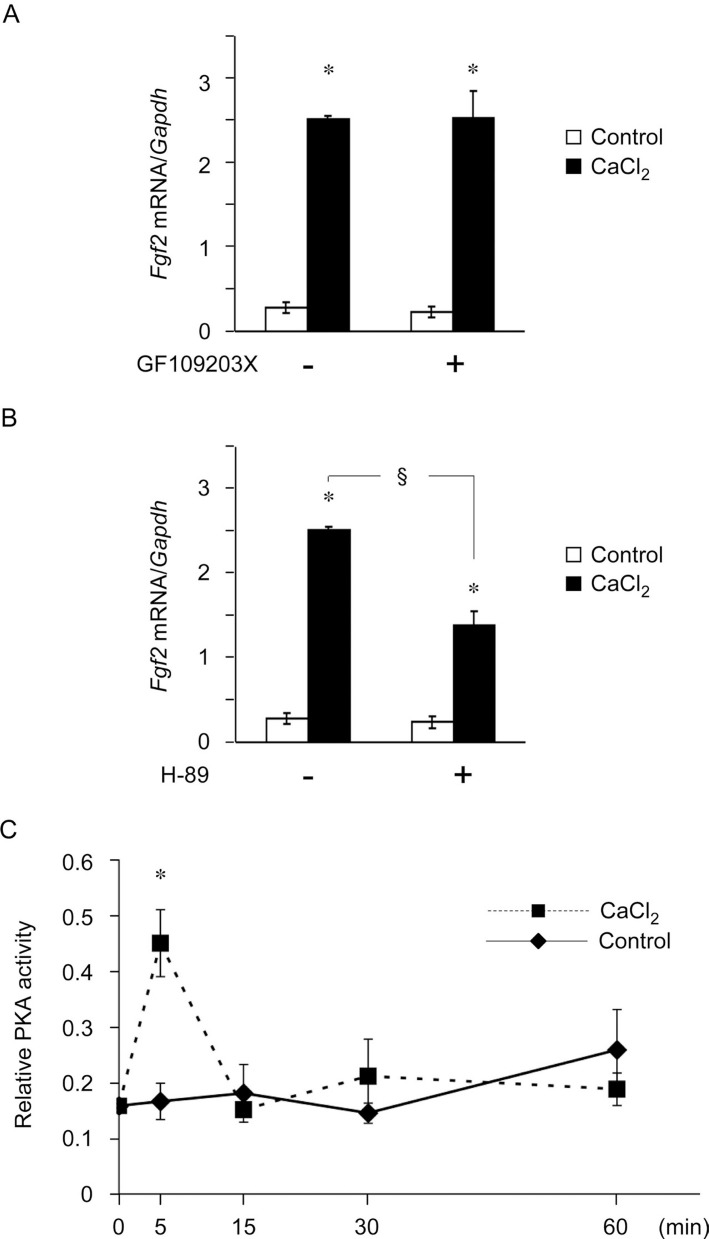
Extracellular Ca^2+^ increased fibroblast growth factor 2 (*Fgf2*) expression via the protein kinase A (PKA) pathway in mDP cells. A subconfluent monolayer of mouse dental papilla (mDP) cells was serum-deprived for 4 h. (A and B) mDP cells were pretreated with the protein kinase C inhibitor GF-109203X or PKA inhibitor H-89 and were then stimulated with 10 mM Ca^2+^ for 6 h in a serum-free medium. *Fgf2* expression was analyzed using real-time reverse transcription-polymerase chain reaction. (C) mDP cells were stimulated with 10 mM CaCl_2_ for the indicated times in a serum-free medium. Cell lysates were collected to measure PKA activity via ELISA. Representative data from three separate experiments are shown as the means ± SD of triplicate assays. *, *P*<0.05 versus untreated cells using analysis of variance; §, *P*<0.05 versus cells treated with 10 mM CaCl_2_ alone using Student's *t*-test.

We determined whether PKA or PKC signaling pathways were involved in CaCl_2_-stimulated *Fgf2* upregulation. As shown in Figure 4A, pretreatment with the PKC inhibitor GF109203X did not inhibit the CaCl_2_-stimulated increase in *Fgf2* expression. By contrast, pretreatment with the PKA inhibitor H-89 significantly inhibited the CaCl_2_-stimulated increase in *Fgf2* expression (Figure 4B). Figure 4C shows that PKA activity was significantly increased after 5 min of stimulation with CaCl_2_; this was followed by a decrease to the control level. These results indicated that the PKA pathway was involved in CaCl_2_-induced *Fgf2* upregulation.

### Extracellular Ca^2+^ increased *Fgf2* expression via ERK1/2 in mDP cells

**Figure 5:**
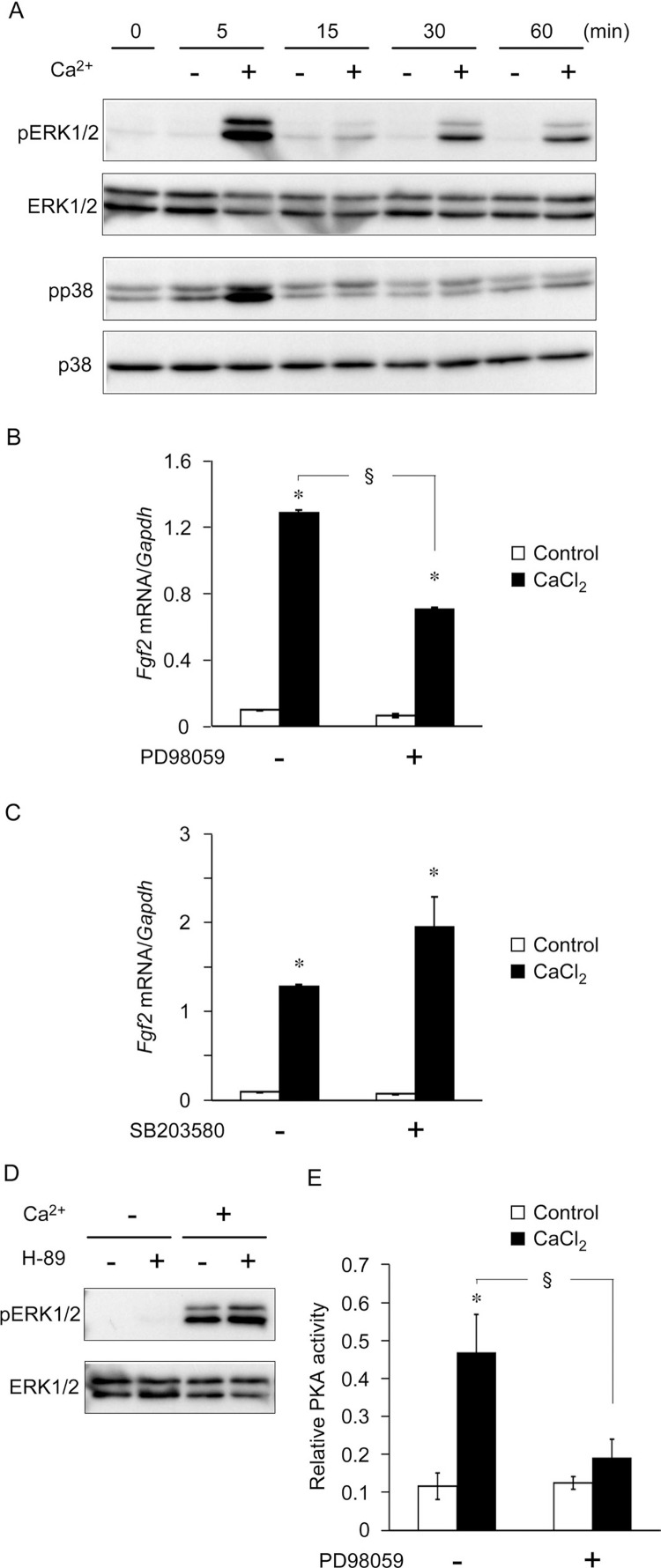
Extracellular Ca^2+^ increased fibroblast growth factor 2 (*Fgf2*) expression via extracellular signal-regulated kinase (ERK) 1/2 and protein kinase A (PKA). A subconfluent monolayer of mouse dental papilla (mDP) cells was serum-deprived for 4 h. (A) Cells were stimulated with 10 mM Ca^2+^ for the indicated times in a serum-free medium, after which cell lysates were analyzed by Western blotting. (B and C) mDP cells were pretreated with the ERK1/2 inhibitor PD98059 or p38 inhibitor SB203580 and were then stimulated with 10 mM Ca^2+^ for 6 h in a serum-free medium. *Fgf2* expression was analyzed using real-time reverse transcription-polymerase chain reaction. (D) mDP cells were pretreated with H-89 and then stimulated with 10 mM Ca^2+^ for 5 min in a serum-free medium. Cell lysates were analyzed by Western blotting. (E) mDP cells were pretreated with PD98059 and then stimulated with 10 mM Ca^2+^ for 5 min in a serum-free medium. Cell lysates were collected to measure PKA activity via ELISA. Representative data from three separate experiments are shown as the mean ± SD of triplicate assays. *, *P*<0.05 versus untreated cells using analysis of variance; §, *P*<0.05 versus cells treated with 10 mM CaCl_2_ alone using Student's *t*-test.

We determined whether mitogen-activated protein (MAP) kinase signaling pathways were involved in CaCl_2_-stimulated increases in *Fgf2* expression. Initially, we examined the phosphorylation status of MAP kinases by Western blotting. Figure 5A shows the biphasic phosphorylation of ERK1/2, with an initial peak at 5 min and a second sustained peak at 30–60 min. The phosphorylation of p38 MAP kinase was detected 5 min after stimulation with CaCl_2_, after which it declined. We then examined the effects of ERK1/2 and p38 on *Fgf2* expression. Pretreatment with the ERK1/2 MAP kinase inhibitor PD98059, but not with the p38 MAP kinase inhibitor SB203580, inhibited the increase in *Fgf2* expression, indicating that ERK1/2 was involved in the CaCl_2_-induced increase in *Fgf2* expression (Figure 5B and C). To examine the interaction of PKA with ERK1/2, cells were stimulated with CaCl_2_ in the presence or absence of H-89 and the phosphorylation status of ERK1/2 was evaluated. Figure 5D shows that CaCl_2_-induced ERK1/2 phosphorylation was not inhibited in the presence H-89. To determine whether ERK1/2 acts upstream of PKA, cells were stimulated with CaCl_2_ in the presence or absence of PD98059 and PKA activity was measured. CaCl_2_-induced increases in PKA activity were inhibited by pretreatment with PD98059 (Figure 5E), indicating that ERK1/2 activated PKA.

## Discussion

In the present study, extracellular Ca^2+^-induced *Fgf2* expression was mediated by ERK1/2 and PKA, but not by PKC, in mDP cells. A previous study revealed extracellular Ca^2+^-induced FGF2 expression via PKA in cementoblasts, which are a highly matured cell type^13^. Conversely, the PKA activator forskolin and the PKC activator phorbol myristate acetate regulate FGF2 expression in osteoblastic MG-63 cells and transforming growth factor beta increases FGF2 expression via PKA in MG63 cells^25^, indicating the existence of multiple signaling pathways that regulate FGF2 expression. ERK also increases FGF2 expression. In a previous study, amitriptyline enhanced FGF2 gene expression via receptor tyrosine kinase (RTK)/ERK/EGR1 in astrocytes^12^. Furthermore, mechanical stress increased FGF2 expression via the PKA and ERK1/2 signal transduction pathways in the MC3T3-E1 osteogenic cell line^15^. However, the relationship between PKA and ERK1/2, which induce FGF2 expression, was not investigated. Figure 5D and E suggest that ERK1/2 activated PKA and mediated extracellular Ca^2+^-induced increases in *Fgf2* expression. PKA and MAP kinase pathways interact with each other. In the PKA pathway, upon activation of a G protein-coupled receptor (GPCR), active Gα subunits activate adenylate cyclase, which generates adenosine 3′,5′-cyclic monophosphate (cAMP). cAMP binds to the regulatory subunits of PKA and induces PKA activation. Crosstalk between PKA/PKC and MAP kinase has been reported^8^. The present study revealed an ERK/PKA signaling pathway induced by extracellular Ca^2+^ in mDP cells. Although cAMP/PKA signaling pathways primarily stimulate RAF/MEK/ERK activity, the MAP kinase cascade can also modulate PKA activity. One mechanism is ERK2-mediated PKA activation via the inhibition of phosphodiesterase (PDE), which hydrolyzes cAMP into AMP. Another mechanism is activation of cytosolic phospholipase A_2_ (cPLA_2_) by ERK, release of arachidonic acid, synthesis and secretion of prostaglandin E_2_ (PGE_2_), and subsequent activation of cAMP/PKA induced by the PGE_2_ receptor^10^. Neurotrophins activate ERK via tyrosine receptor kinase B and transiently inhibit PDE, leading to elevated cAMP levels and nerve regeneration^7^. Platelet-derived growth factor induces ERK, the phosphorylation of cPLA_2_, the rapid release of PGE_2_; as a consequence, cAMP generation is induced in human arterial smooth muscle cells^9^.

Osteoblasts express CaSR, which is a GPCR first cloned from the parathyroid gland^3^, and CaSR promotes osteoblast proliferation and differentiation and bone formation^29^. In addition, another extracellular calcium-sensing GPCR, GPRC6A, which is functionally similar, but molecularly distinct, from CaSR, is involved in the modulation of osteoblast differentiation^22^. In addition to GPCRs, RTK possesses a calcium-sensing mechanism. FGF receptors have an extracellular acidic box region with high affinity for Ca^2+^ and regulate cellular function^19^. Our previous studies demonstrated that extracellular Ca^2+^ influx via Ca^2+^ channels is not involved in CaCl_2_-mediated FGF2 regulation in cementoblasts^13^ and that CaCl_2_-stimulated *BMP2* expression requires the ERK pathway and Ca^2+^ influx from L-type Ca^2+^ channels in hDP cells but that the phosphorylation of ERK does not require Ca^2+^ channels^26^. We were unable to detect the calcium-sensing mechanism involved in extracellular Ca^2+^-induced FGF2 and *BMP2* expression in murine cementoblasts and hDP cells and speculated that a distinct putative receptor is expressed by cementoblasts and hDP cells because of the different ligand specificities of Ca^2+^ as well as divalent and polyvalent cations^13^ ^,^ ^26^. In the present study, CaSR and GPRC6A gene expression in mDP cells was not detected, indicating that a different calcium-sensing mechanism, such as RTK or GPCR-mediated RTK transactivation^6^, is involved in extracellular Ca^2+^-induced *Fgf2* expression in mDP cells.

## Conclusion

Elevated extracellular Ca^2+^ levels led to increased *Fgf2* expression in mDP cells through PKA and ERK1/2. Extracellular Ca^2+^ simultaneously increased the gene expression of FGF2 and BMP2. However, the effects of FGF2 and BMP2 on dental papilla cells are unknown. Further investigations of the roles of FGF2 and BMP2 in dental papilla cells are required. Our findings may contribute to designing regenerative therapies for dentin on the basis of reliable biological principles.
